# *“Ten years of war! You expect people to fear a ‘germ’?”*: A qualitative study of initial perceptions and responses to the COVID-19 pandemic among displaced communities in opposition-controlled northwest Syria

**DOI:** 10.1016/j.jmh.2020.100021

**Published:** 2020-12-07

**Authors:** Yazan Douedari, Mervat Alhaffar, Muhammed Al-Twaish, Hala Mkhallalati, Raheb Alwany, Nafeesah Bte Mohamed Ibrahim, Ayshath Zaseela, Nour Horanieh, Aula Abbara, Natasha Howard

**Affiliations:** a Syria Research Group (SyRG), co-hosted by the London School of Hygiene and Tropical Medicine and Saw Swee Hock School of Public Health, United Kingdom; b London School of Hygiene and Tropical Medicine, Department of Global Health and Development, 15-17 Tavistock Place, London, WC1H 9SH, United Kingdom; c Hand in Hand for Syria/Yardim Uzmanlari Yardimlaşma ve Kalkinma Derneği, Idlib, Syria; d Faculty of Population Health, Institute for Global Health, University College London, 30 Guilford Street, London, WC1N 1EH, United Kingdom; e NHS Foundation Trust, York Teaching Hospital, Wigginton Rd, Clifton, York YO31 8HE, United Kingdom; f Imperial College London, Department of Infectious Disease, St Mary's Hospital, Praed Street, W2 1NY, United Kingdom; g National University of Singapore, Saw Swee Hock School of Public Health, 12 Science Drive 2, 117549, Singapore

**Keywords:** Displacement, Conflict, Lived experience, COVID-19, Syria

## Abstract

**Background:**

Response to the COVID-19 pandemic has challenged even robust healthcare systems in high-income countries. Syria, a country experiencing protracted conflict, has the largest internally-displaced population globally with most displaced settlements in opposition-controlled areas governed by local and international NGOs. This study aimed to explore community perspectives on challenges and potential solutions to reduce COVID-19 transmission among displaced communities in opposition-controlled Northwest Syria.

**Methods:**

We used a qualitative study design, conducting 20 interviews with displaced Syrians in opposition-controlled camps in Northwest Syria between April-May 2020 and ensuring over half our interviewees were women. We analysed data thematically.

**Results:**

Participants described already difficult camp conditions that would be detrimental to an effective COVID-19 response, including household crowding, inadequate sewerage and waste management, insufficient and poor-quality water, and lack of cleaning supplies. Participants most frequently mentioned internet as their COVID-19 information source, followed by NGO awareness campaigns. Men had access to more accurate and comprehensive COVID-19 information than women did. Isolating (shielding) high-risk people within households did not appear feasible, but participants suggested ‘house-swapping’ approaches might work. While most participants had sufficient knowledge about COVID-19, they lacked practical tools to prevent transmission.

**Conclusion:**

This study is the first to explore perspectives and lived experiences of internally-displaced Syrians in the weeks prior to the COVID-19 epidemic in Northwest Syria. The challenging living conditions of internally-displaced people in Syria are further threatened by the spread of COVID-19. Tailored control measures are urgently needed to reduce COVID-19 transmission in camps.

## Background

### COVID-19 concerns for conflict-affected countries

The COVID-19 pandemic has resulted in over 61 million cases and 1.4 million deaths at the end of November 2020 (Coronavirus Cases 2020). Responding to the pandemic has challenged even robust high-income healthcare systems and remains a devastating threat to many low and middle-income countries, especially those affected by conflict. However, much of the published literature on COVID-19 is from high-income countries, with less attention given to countries with weaker healthcare systems (Hariri et al., 2020). With many high-income countries struggling to provide effective responses, including mass testing and physical distancing, conflict-affected countries such as Syria face additional challenges. The impact of COVID-19 on displaced populations, particularly internally displaced people (IDPs) in fragile and conflict-affected settings, remains poorly understood (Shammi et al., 2020). IDPs do not have the same legal protections that international refugees have, with a tenuous status often reliant on the very authorities that caused their flight (Orendain and Djalante, 2020). IDPs are also more likely to have inadequate shelter, with some sheltering in camps, schools, or shared and rented accommodation. Displacement camps, which are prone to crowding and poor infrastructure, may worsen COVID-19 risks and outcomes compared to other settings. Globally, of 50.8 million IDPs at the end of 2019, 45.7 million were displaced due to conflict violence (IDMC 2020). There are mounting concerns that IDPs in low-income countries could experience particularly severe COVID-19 cases and related deaths (Orendain and Djalante, 2020, Abbara et al., 2020).

### Conflict and IDPs in Syria

The largest IDP population globally is in Syria (Internally Displaced People -. UNHCR Syria 2020), with 6.5 million displaced in a population of 17 million (Syria. IDMC 2020). Displacement is a result of almost a decade of armed conflict, which has also decimated Syria's health system and divided it among several fragmented areas of military control (Douedari and Howard, 2019). The opposition-controlled area (OCA) in Northwest Syria hosts an estimated 4.2 million people, over 2.8 million of them IDPs from across the country (WHO 2019, Mobility And Needs Monitoring | Syrian Arab Republic 2020). IDP camps in OCA shelter an estimated 1.5 million displaced Syrians - over 75% of whom are women and children - and are governed by local and international NGOs, while some are self-governed by camp residents or not governed at all (Syrian Arab Republic | ReliefWeb 2020). UN agencies do not operate directly in IDP settlements, but rather provide support to NGOs. IDP settlements face various challenges, including overcrowding, inadequate food, insufficient clean water, and weak healthcare infrastructure (Marzouk et al., 2020).

Overcrowding is common, with an estimated 327,000 IDPs in tents sheltering 6-12 people each (Abbara et al., 2020). Displacement, lack of livelihoods, and inadequate food security increased IDP dependency on humanitarian organisations such as the World Food Programme, which provides food assistance to 4 million food-insecure people in Syria, including in OCA (FAO 2019). Insufficient clean water is due to contamination from deteriorating infrastructure and wastewater flooding during ongoing conflict (ReliefWeb 2020). Sanitation is of particular concern in OCA, in which growing IDP settlements share community latrines that do not meet minimum humanitarian standards (ReliefWeb, 2020).

Weak healthcare infrastructure is due both to indirect consequences of the conflict (e.g. lack of staff and supplies) and direct attacks. As of April 2020, only 306 of 568 OCA health facilities remained functional (Humanitarian Response 2020). Thousands of health-workers have left Syria, and despite estimates we do not know the true numbers (Physicians for Human Rights 2020). At least 923 were killed in 595 attacks on 350 separate health facilities since the conflict began, approximately 90% committed by the Syrian regime or its allies (Physicians for Human Rights 2020). Consequently, health facilities and health-workers were unable to meet population needs even prior to the COVID-19 pandemic reaching Syria (Kassem, 2020).

### COVID-19 preparation and response in opposition-controlled northwest Syria

Many challenges hinder IDP capacity to respond to COVID-19 risks. Physical distancing and self-isolation, which can reduce virus transmission (Colbourn, 2020, Chu et al., 2020), are virtually impossible in crowded IDP settlements (Blumenthal and Murdoch, 2020, Kassem and Jaafar, 2020). Dependency on provisions from humanitarian organisations hinders IDPs’ ability to restrict movements during any lockdowns (Asseburg et al., 2020). Insufficient WASH (water, sanitation, and hygiene) facilities, particularly in camps or collective shelters, make frequent hand-washing largely impossible (The New York Times 2020). Insufficient health facilities and health-workers in IDP settlements exacerbate these prevention challenges by reducing opportunities to detect and treat symptomatic individuals. However, some examples of community-led engagement exist, focusing on addressing COVID-19 misinformation and supporting communities within local governance structures (Ekzayez et al., 2020).

Testing capacity and implementation remain major concerns, as, for example, OCA had only one testing laboratory with a capacity of 100 tests per day for a population of 4 million at the beginning of the pandemic (Syrian Arab Republic | ReliefWeb 2020, Al Jazeera 2020). OCA Health Information System Unit modelling, forecasting the possible impact of COVID-19 on its camp-population, predicted 240,000 cases (20% of camp residents) in the first 6 weeks of which 36,000 would be severe, 12,000 critical, and 14,328 would result in death (Hariri et al., 2020). With only 2429 inpatient beds and 240 intensive-care beds (i.e. 98 adult ventilators, 62 paediatric ventilators), OCA capacities could quickly be overwhelmed (Hariri et al., 2020). To better support COVID-19 prevention among displaced people in Syria, we need to know more about their lived experiences and perceptions of potential prevention and mitigation measures.

### Objectives

This study aimed to explore challenges and potential solutions to reducing the spread of COVID-19 in IDP settlements in opposition-controlled Northwest Syria. Objectives were to: (i) identify existing living conditions that could challenge COVID-19 prevention efforts; (ii) describe community knowledge and preparations for COVID-19, including perceptions about common protective measures against COVID-19 transmission; and (iii) document any promising COVID-19 mitigation suggestions for camp-based communities. Implications for policymakers and practitioners implementing community-level measures to mitigate COVID-19 in IDP camps are discussed.

## Methods

### Study design and methodological orientation

We chose a qualitative study design, informed by an interpretative phenomenological approach as described by Smith *et al*, using semi-structured remote and in-person key informant interviews with adult men and women living in IDP camps in opposition-controlled Northwest Syria.

### Research question

Our research question was: “How do displaced community members describe COVID-19 risk and prevention responses in IDP camps prior to the pandemic's arrival in Northwest Syria?”

### Participant selection

We used a two-stage sampling process, as recruitment was challenged by confidentiality and safety concerns, limited electricity/internet access, and time constraints. First, we sampled personal WhatsApp network contacts purposively to provide a broad range of perspectives. This provided 14 participants. Second, we snowballed from each contact by asking them to identify two other potential participants, giving 6 participants. We ensured that at least half of participants were women, both to counter Syrian women's frequent underrepresentation in research and because women were more likely to know the information we were examining. Twenty-one people declined to participate due to safety concerns or unfamiliarity/discomfort with research interviews.

### Data collection

We developed an Arabic topic guide, based on the literature and expert consultation, to examine IDP living conditions, access to COVID-19 related information, prevention strategies, experiences of lockdown and any behaviour changes during Ramadan (Muslim holy month of fasting), while allowing exploration of emerging concepts. The topic guide was piloted in two interviews and refined iteratively. Interviews averaged 35 minutes each. As participants were based in Syria, and authors in the United Kingdom and Syria, most interviews were conducted using WhatsApp freeware (a cross-platform messaging and Voice-over-IP application) and some were conducted in-person while maintaining COVID-19 physical distancing in accordance with WHO guidelines (World Health Organization 2020).

YD, MT, and HM conducted interviews in April-May 2020 (Table 1). To obtain informed consent, we sent potential participants electronic copies of study information sheets and consent forms via WhatsApp and arranged individual meetings to address questions and concerns. We then recorded verbal consent, for those willing to participate, prior to interview. We did not attempt written consent, as: (i) remote participants could not print consent forms and they were too challenging to complete on their phones; and (ii) face-to-face interviews needed to be physically distanced to reduce COVID-19 transmission risk.

**Table 1:** Participant details.

ID	Gender	Camp location	Shelter type	People sharing	Shared toilet
CR9	Man	Hezreh (Al-Dana)	Tent	9	Yes
CR11	Man	Hezreh (Al-Dana)	Tent	6	Yes
CR20	Woman	Hezreh (Al-Dana)	Tent	6	Yes
CR12	Man	Hezreh (Al-Dana)	Concrete with fabric ceiling	8	No
CR13	Man	Jesr Al-Shoghour	Tent	6	Yes
CR19	Woman	Mashhad Al-Rawheen	Tent	10	Yes
CR6*	Woman	Sarmada	Tent	15	No
CR7*	Woman	Sarmada	Tent	12	Yes
CR1*	Woman	Sarmada	Two-room tent	22	No
CR2*	Woman	Sarmada	Tent	10	Yes
CR3*	Man	Sarmada	Tent	10	Yes
CR5*	Woman	Sarmada	Tent	12	Yes
CR4*	Woman	Sarmada (Tiba)	Caravan	10	No
CR14*	Woman	Sarmada (Tiba)	Caravan	4	No
CR15*	Woman	Sarmada (Tiba)	Caravan	2	No
CR8	Man	Termaneen	Flat	4	No
CR10	Man	Termaneen	Concrete with fabric ceiling	5	No
CR16	Woman	Termaneen	Flat	4	No
CR17	Woman	Termaneen	Flat	4	No
CR18	Woman	Termaneen	Flat	7	No

⁎ NB: Indicates interviews conducted in-person.

We conducted interviews at times chosen by participants, digitally recorded them with participant consent, and took extensive notes in Arabic to contextualise findings. Interviews were recorded anonymously using numerical identification codes and transcribed by the team. We stored audio recordings, transcripts, and notes in password-protected files on LSHTM servers. An on-call Arabic-speaking psychotherapist was available to provide remote psychological support for participants and interviewers who needed it, though none used this support.

### Analysis

We determined data saturation using a saturation grid as described by Fusch and Ness (2015). YD, MT, and HM analysed data thematically in Arabic according to the six phases described by Smith *et al*: (i) reading and re-reading; (ii) initial noting; (iii) developing themes; (iv) searching for connections across themes; (v) moving to the next case; and (vi) looking for patterns across cases. We developed and connected themes using a combination of abstraction, subsumption, and polarisation, contextualising our themes according to question guide topics and extensive notes taken during interviews. Relevant quotes were translated to English by bilingual co-authors. Themes were reviewed by NH and discrepancies agreed between investigators. Reporting adheres to COREQ criteria (Tong et al., 2007).

### Reflexivity

Interviewers, 2 men and 1 woman, are current or former Syrian health-workers with in-depth knowledge of the Syrian socio-political context, and participants responded to them as such. This appeared to facilitate trust, willingness to participate, and discussion. Interviewers had experience conducting qualitative interviews in Syria. YD and HM have MSc degrees in public health and MT has an MSc degree in orthodontics, which influenced their interpretation. All interviewed women, while only men interviewed men, though no differences were noted in women's responses and COVID-19 was not considered a gender-sensitive topic in the Syrian context. No interviewers had personal relationships with any participants.

### Ethics

We received ethics approval from the Observational Research Ethics Committee at the London School of Hygiene & Tropical Medicine (reference 17360).

## Findings

### Participant characteristics

We conducted 20 interviews, 11 by WhatsApp and 9 in-person (Table 1). Thirteen participants (65%) were women. Nine participants lived in Sarmada, 5 in Termaneen, and 4 in Hezreh, 13 accommodated in tents or tent-like structures, 4 in apartments, and 3 in caravans. The average number sharing each accommodation was 8.3 people with almost half (9 households) sharing toilet facilities with other households.

### Thematic analysis

Findings are presented under five themes: (i) living conditions that could affect COVID-19 prevention; (ii) COVID-19 prevention knowledge and perceptions; (iii) individual and household COVID-19 prevention behaviours; (iv) official responses to COVID-19 risk; and (v) effects of COVID-19 risk and responses on daily life.

### Living conditions that affect COVID-19 prevention

Camp conditions that participants described as particularly detrimental to an effective COVID-19 response were household crowding, inadequate sewerage and waste management, insufficient and poor-quality water, and lack of cleaning supplies.

*Household crowding.* Over half of participants had been displaced in their camp since conflict began in 2011, while the rest had been displaced multiple times (e.g. 4-5) before arrival. Investigators observed that length of displacement appeared to correspond with better living conditions. For example, those displaced longest lived with immediate family (e.g. spouse and children) or extended family (e.g. parents, siblings), while more recent arrivals often shared a household with 1-2 other families. The half of participants who arrived earlier generally lived in 9-24m^2^ shelters with private kitchen, toilet, and shower. These were either moveable fibreglass ‘caravans’ or 1-2 room one-story detached concrete shelters with solid or tent-fabric roof. These shelters were described as better than tents. Participants who arrived later generally lived in 4 × 4m^2^, 4 × 6m^2^, or 4 × 9m^2^ tents and shared sex-segregated public toilet and shower blocks distributed across the camp. As one participant described:

“We have 260-270 tents in the camp here. In each tent there is a family. Each family is 3-11 persons. We have 4 toilet blocks. Each block has 2 male toilets and 2 female toilets. We have plenty of issues with toilets. In most occasions you see people queuing, very crowded. One might stand for 15-30 minutes in the queue…” CR11

*Inadequate sewerage and waste management.* Participants reported that disposal was not properly managed within camps, with one describing sewage rivulets running between tents. Participants expressed concerns about the risk of disease spread due to inadequate hygiene. Participants blamed NGOs for inconsistent waste management and not resolving waste disposal issues, despite multiple requests, thus forcing camp residents to try unsuccessfully to dispose of waste themselves.

“The toilets and showers are so dirty and not hygienic at all. We cannot shower there. I clean myself in my tent and so does the rest of my family… Some people are digging their own toilets, in a very bad manner, which makes all the sewage waste always overflow.” CR17

*Insufficient water.* Half of participants reported accessing shared chlorinated water tanks, refilled irregularly by NGOs. Others reported having household water tanks, either provided by NGOs or purchased independently. NGOs sometimes provided drinking water and sometime participants had to buy non-chlorinated water. Participants reported boiling water before drinking it, while one described having to buy expensive filtered drinking water as she suffered from kidney problems. Participants described water quality as a major concern, with some reporting dirt and debris in the water and others describing skin irritation, possibly from over-chlorination.

“It comes out of the tap dirty and undrinkable. We use it for bathing and it causes us rashes...” CR20

*Insufficient cleaning supplies.* All participants highlighted that they could not get sufficient cleaning materials as they were too expensive. Some reported receiving NGO hygiene kits in a one-time distribution. Participants said they bought only basic cleaning products, such as soap and shampoo, and much less than they bought before displacement so they sometimes ran out of cleaning supplies.

“If I tell you we are getting enough [cleaning] material, I would be lying to you. We are barely able to get soap and in best cases a shampoo. It is difficult to get disinfectants like chlorine products, we are not even thinking of them because there are other things that we need like food” CR12

### COVID-19 prevention knowledge and perceptions

Participants described a range of information sources, reasonably accurate knowledge that differed by sex, and perceptions of the virus, challenges of isolating high-risk and potentially infected household members, and risks of imposing curfews or lockdowns.

*COVID-19 information sources.* Participants most frequently mentioned television and internet as COVID-19 information sources, followed by NGO awareness campaigns inside or outside camps. The few participants who did not have internet or television access relied on information circulated by their neighbours and relatives. Major internet sources were Facebook, WhatsApp, YouTube, Telegram, and well-established media outlets such as the BBC, France 24, and Aljazeera. Facebook was the most mentioned information resource.

“[I get information] from the internet mostly, because we don't have electricity [for TV]. Among every 2-3 households, one would have electricity and neighbours would come and charge their [phone] batteries […] so we don't have TV. We get all news through our mobile phones” CR10

*Reported COVID-19 knowledge.* Approximately half of participants reported accurate information on COVID-19 prevention, isolation, hygiene practices, and shielding high-risk people. Investigators noted a correlation between gender and information provided, with men having access to more accurate and comprehensive information. This was potentially due to awareness campaigns being directed towards men and gender-differentiated Syrian socialisation norms. Several participants provided some misinformation when asked about COVID-19 transmission, prevention, and high-risk people. Examples included expecting certain and rapid death after infection, all ages being affected similarly, and inaccurate hygiene techniques such as only washing with water (without soap). Several were unable to identify high-risk household members. Many stated that they would seek immediate help from health facilities if they contracted the virus, which contradicted local guidelines advising symptomatic patients to self-isolate if able to cope.

“The first thing I would do is take them [infected people] to the hospital where they can be treated” CR16

*Perceptions of COVID-19.* Several expressed disbelief that COVID-19 would reach their area and affect their families. It was described as ‘too unfair’, or ‘too bad to be true’, given their already challenging situation.

“To be honest, it's not only me who is not committed to infection prevention measures. Everyone here in the camp is the same. We believe, especially after being internally-displaced and everything else we went through, that this virus is not going to hit us...” CR16

Several participants indicated that coronavirus itself was not their main concern. Their main concerns were having better infrastructures, particularly improved sewerage and waste management, access to clean water, and private toilets and bathing facilities. Most stressed that facilitating livelihoods and better economic opportunities were crucial for people in the camps.

“People in the camps have much more pressing issues to think of. We need to work and get money. We need clean water.” CR17

*Perceptions of isolating high-risk or infected individuals.* Participants suggested isolating high-risk household members would be challenging due to camp overcrowding, small shelters, and communal living.

“If someone in the camp gets infected, we either isolate them in collaboration with the camp community, or I would move our tent to a distant place in the mountain.” CR13

One participant suggested providing services for high-risk individuals to reduce their need to go out in crowds, for example buying their groceries. Another participant suggested that exchanging tents to make room for high-risk individuals would be possible.

“We are all family and relatives here [in the camp]. If we had to we could vacate one tent for them [high-risk individuals] and the rest of us share one tent with each other” CR11

Most participants indicated that they would self-isolate in a separate tent away from the camp if they suspected they were infected with coronavirus and would use separate items like utensils. However, given the challenges in obtaining shelters in camps this solution appeared difficult or unlikely (e.g. only for those able to afford to buy another tent or a ‘tent’ might mean stringing together any available fabric as an inadequate tent-like shelter). Several participants indicated that they would direct suspected COVID-19 cases to health facilities to be isolated there, as this was the health facility's responsibility. All participants reported that self-isolating within the household shelter was impossible.

“I can separate their [potentially-infected household member] plate, spoon, and glass. However, there is no way in this one-room house to get them isolated in a 2 × 2 meter area”*CR18*

*Perceptions of lockdown and curfews.* Participants discussed the possibility of lockdown (i.e. a broad emergency measure, closing private and public spaces and keeping people home as much as possible but maintaining essential services) or curfew (i.e. a narrower administrative order, imposed frequently such as during riots or violence in specified hours and areas, to keep people home and public spaces and essential services such as markets and schools closed for a set number of hours). Participants did not distinguish between lockdown and curfew in their responses, instead highlighting challenges related to implementation of either measure and questioned their feasibility in camps given existing overcrowding, shared toilets and showers, and the crucial absence of enforcement bodies.

“Two or three members of each household go out to work daily to provide for their families. How would you impose curfew? It would be a crime!” CR13

Participants expressed particular concern about potential loss of already limited incomes and food aid in the case of lockdown. As work could not be done remotely, food aid required queuing, and food storage was not feasible in camp shelters.

“[Lockdown] would be difficult. I mean, if it is applied to all people and people have to do it, then they will. But then how will people eat? How will they get money to survive?” CR19

Participants used words such as “starve”, “survive”, or “all the money I have” to accentuate the threat of extended curfew or lockdown.

“If we sit at home, we will starve to death” CR10

Several participants indicated the possible positive prevention effects of lockdown, if implemented correctly by allowing essential services such as food aid for those in need. However, even these participants indicated this would be difficult in camps.

“The curfew might be good to solve and overcome the coronavirus problem, but it has a strong negative impact in terms of poverty and hunger. If we stay home we're not relieved, and if we go out to work we're not relieved.” CR13

While effects of school closures were not discussed separately from other forms of lockdown, as children in camps either attended unofficial camp ‘schools,’ formal schools in nearby villages, or worked to help their families survive without any additional education. Given existing educational constraints, only one participant highlighted the detrimental effects of school closures on children and young people in camps and that online education was not readily accessible, as worsened access was largely hypothetical*.*

### COVID-19 prevention behaviours

Several participants reported they had adopted COVID-19 prevention measures, including handwashing, physical distancing, staying home as much as possible, avoiding crowds, and avoiding shaking hands. One participant reported replacing home visits with internet communications and another reduced shopping.

“We don't go to the market unless it is exceptionally necessary. We limit our market visits to once or twice per week” CR10

Some participants suggested improving nutrition and immunity, and ensuring regular medication for high-risk people, for COVID-19 prevention. A few reported increasingly obsessive hygiene practices, such as washing vegetables up to 10 times. Some described how their own behaviour had changed while others’ had not.

“Both me and my husband have asthma. I also have kidney failure and diabetes. My husband is getting so scared. He bought us antiseptics and disinfectants. We have limited our contact with people. Others in the camp, however, are not cautious at all. They say there is no such a thing as Coronavirus, at least in our area.” CR20

Approximately half indicated that they had not changed their behaviour because there was no point.

“Do you know why? Because we left the most precious things in life, our homes. We were evicted from our homes. We don't have anything left. Nothing matters anymore” CR9

### Official responses to COVID-19 risks

*Local authority responses*. Authority is very fragmented in the area, and could be Salvation government, camp management, or health directorates. Local authorities initially closed borders, mosques, and large markets in occupation-controlled areas, though mosques and markets were reopened after a month. Administrators in one camp banned travelling salespeople as a potential source of infection from outside the camp and between multiple camps and villages. They similarly tried to limit social contacts by advising people to reduce movement inside and outside camps. A few participants indicated they had not noticed any COVID-19 related changes.

“We did not feel any change, as our camp is isolated and we have no contact outside the camp” CR13

*NGO responses*. Participants reported being told to maintain a one-metre distance when queuing, reduce gatherings, increase disinfection, and wear masks. They described humanitarian NGO-led COVID-19 awareness campaigns.

“All people used to gather [for aid distribution], now they [NGO workers] are wearing masks and putting an insulator. Children are not allowed. People come and collect [aid] without gathering. Each name is called upon to collect” CR2

No specific help was reported at the time of interviews in implementing distancing and hygiene requests (e.g. free reusable mask distribution, hygiene kits).

“Nothing really. We started to get aid, and while we waited on the queue, they asked us to keep a meter distance between each other...” CR17

Only one reported food aid being delivered instead of residents being required to queue for collection.

“Bread is being delivered to caravans now, while people had to go and collect before” CR4

*Health facility responses.* Participants described seeing increased disinfection procedures in health facilities, including health-workers continuously wearing masks, fewer gatherings, and opening of two quarantine centres in the region.

### Effects of COVID-19 on daily life

As cases had not yet been reported in these camps at the time of our interviews, effects on daily life were either anticipated or due to preparatory behavioural and environmental changes. Therefore, participants expressed a combination of fears, dismissal, and resignation depending on the changes already enacted in their camps. Discussion focused on income and expenses, expectations for Ramadan, and psychological distress.

*Income and expenses*. Some participants described reduced or lost work income due to COVID-19 especially that demand for labour decreased with border closures. Reported price inflation of essential goods were major concerns for several participants.

“Nothing has changed except for the prices, which got higher and higher because of the closed borders.” CR19

*Ramadan*. Participants had mixed experiences of the holy month during the COVID-19 pandemic. Some reported decreased felt spirituality due to reduced or no communal prayers in mosques and uncertainty about mosque closures and risks of attendance. Participants were particularly concerned *Tarawih* prayers (additional night-time prayer during Ramadan) might need to be performed at home. Several highlighted a potentially preventive effect on COVID-19 transmission as movement outside home naturally decreased during Ramadan. However, some reported no observable changes.

“Life in the camp is normal. Children are playing with each other, men are drinking tea together outside tents, and Tarawih prayer is ongoing” CR13

*Psychological distress.* Some reported emotional signs of stress, including increased fear and worry, insomnia, nightmares, and sadness.

“Even though the disease has not reached the area yet, we are living in fear and terror… because we are living in camps” CR10

Stress responses varied, however, with one participant articulating the implicitly pragmatic risk prioritisation common within camps.

“Ten years of war! You expect people to fear a germ? Everything became acceptable. Even death is acceptable” CR14

## Discussion

### Study contribution

This study is unique in exploring perspectives and lived experiences of internally-displaced Syrians in the weeks prior to the COVID-19 epidemic in Northwest Syria. This focus on voices of the displaced is particularly important as they are often missing in public health research in conflict-affected settings such as Syria. This study is also distinctive in that it is led by current and former Syrian health-workers with insider knowledge of the Syrian conflict and health systems.

### Key findings

Camp residents identified their living conditions as a major existing concern, worsened by the threat of COVID-19. Many lived in crowded one-room tents, sharing unsanitary public toilets and showers, severely constraining their ability to practice either safe-distancing or hygiene-control measures recommended globally to prevent COVID-19 transmission (World Health Organization 2020). This was exacerbated by shortages of water and cleaning supplies. Similar concerns were reported in refugee camps in Bangladesh (Truelove et al., 2020, Vince, 2020) and Lebanon (Kassem and Jaafar, 2020). Risks of large outbreaks spreading from camps was a frequent discussion point, though evidence of this remains limited.

IDPs’ use of social media as COVID-19 information source was supported in the literature (Amsalem et al., 2020, Pennycook et al., 2020), with Pennycook et al. stressing the significant contributions of false information on social media platforms to public misconceptions about the pandemic. Our findings that some participants could not identify high-risk groups or recommended actions contradicting WHO guidelines suggested uncritical acceptance of social media information among at least half of IDPs interviewed. This is important because epidemic preventive behaviours are linked with access to sufficient accurate information (Funk et al., 2009). The significant gender disparity in information accuracy we noted, with women being less aware than men about COVID-19 transmission, prevention, and high-risk groups, may correspond with a 2003 UNESCO declaration deeming lack of access to information ‘the third major challenge’ confronting women in low-income countries, after poverty and violence. However, it contradicts Howard et al's finding that Afghan men shared health information and does not fully fit the Syrian context as many women were well-educated prior to the war and are as capable as men of accessing accurate information. Further research is needed to explore this.

Isolating (shielding) high-risk people within the household shelter, as suggested in proposed guidance by Favas *et al,* does not seem feasible in camp settings based on participant accounts (Favas et al., 2020). However, participants suggested that an approach similar to Favas *et al*’s ‘house-swapping’ was potentially feasible. Similarly, social care could involve the assistance discussed by participants or potentially expanded to general governance of the isolation process as proposed by Favas et al. (2020). Participants similarly indicated they planned to self-isolate in a separate tent if infected, though isolating suspected COVID-19 cases would likely be difficult if widespread disease occurred and large numbers of additional tents were required (Vonen et al., 2020, Alkarim et al., 2020).

The risk of lost household income due to COVID-19 responses such as lockdowns was reported in media and agency reports (Małachowska et al., 2020, Kluge et al., 2020). For example, many refugees in Jordan reportedly lost employment due to lockdown (Dhingra, 2020). Relatedly, the world's largest economies have been badly affected by the COVID-19 pandemic and will likely reduce their humanitarian contributions for displacement settings (Jones et al., 2020, Romei and Burn-Murdoch, 2020). Food aid, a concern among study participants, is reliant on donations from wealthy governments suggesting this was a particularly valid fear.

Indifference to and denial of the pandemic may have related to IDPs lack of felt agency or more urgent priorities, and was also described among refugees in Jordan (Małachowska et al., 2020). Reported psychosocial distress due to COVID-19 was consistent with Gao *et al*’s findings of anxiety and depression among Chinese communities (Gao et al., 2020). Similarly, Kochhar *et al* found mental health concerns were related to isolation and income loss from lockdown (Kochhar et al., 2020). The suggested benefit of Ramadan in reducing interactions and thus COVID-19 transmission due to people's low energy levels was mentioned elsewhere but lacks evidence (Reza et al., 2020).

### Implications for policy and practice

An immediate implication for decision-makers is the likely benefit of developing an action plan to prevent COVID-19 outbreaks in camps. This would help donors and implementing agencies to estimate additional needs for physical distancing, including alcohol-based hand gel and soap for handwashing, masks, and isolation tents. COVID-19 health education should target both women and men. Further research is needed to determine why knowledge levels appeared different, but a gendered approach should be adopted emergency risk communication and humanitarian responses to ensure access for all and rapid correction and responses to widely circulating misinformation (Communicating risk in public health emergencies 2020, Lafrenière et al., 2019, Inter-Agency Standing Committee IASC 1999).

While awareness campaigns are useful, most participants already had sufficient knowledge about COVID-19 but lacked the practical tools to prevent transmission. Specific outbreak planning is needed to reduce potential COVID-19 transmission in camps. This could include distributing an extra tent for each household to shield at-risk household members of isolation the potentially infected, provision of food supplies to those isolating or during lockdowns or peak periods, and storage of stocks of essential items close to camps in case of lockdowns. Resources for dealing with chronic illnesses, mental health, and psychosocial distress should be provided. Additionally, innovative ways to substitute income loss should be explored such as cash-for-work projects (Doocy et al., 2006, Carruth and Freeman, 2020).

Longer-term, humanitarian agencies need to address existing WASH challenges by providing safe water, adequate sanitation (e.g. increasing numbers of toilet and shower blocks where feasible) and sewerage, as these pose serious health risks beyond the current pandemic.

### Future prospects

After completion of our interviews in May 2020, the first cases of COVID-19 in opposition-controlled Northwest Syria were declared on 9 July 2020 and cases have escalated rapidly since. As of the 1 December 2020, over 16,289 cases and 166 deaths have been reported (EWARN, 2020), with many more likely undiagnosed due to testing capacity and logistical challenges. The EWARN-led public health response is focused on testing and contact-tracing, with laboratory numbers increasing to 3 and elevating testing capacity from 100 to 1700 tests daily. However, community circulation of SARS-CoV-2 virus is apparent outside and inside displacement camps (EWARN, 2020). Cases in camps were initially slow to rise, likely due to challenges accessing healthcare and testing by camp residents, and are now increasing more rapidly. Further research examining changing community perceptions and challenges, to inform contextually-relevant interventions, is urgently needed given increasing cases and deaths.

### Limitations

This study had several limitations. First, MT and HM were relatively new to remote qualitative data collection and analysis so some nuances may have been missed. This was mitigated by review of initial transcripts, iterative development of the question guide, and regular co-author discussion. Second, differing living conditions between camps and between older and more recent camp residents could not be fully captured and findings should not be generalised to all OCA camps or camps in other areas of Syria. However, similar difficulties of overcrowding, poor hygiene, and challenged livelihoods are reported in most camps. It should also be noted that, as this study was intentionally conducted prior to the first COVID-19 cases being confirmed in OCA, participant perceptions could have changed since. Similarly, this study intentionally did not include decision-makers who could have very different perceptions to those of camp residents.

## Conclusions

Displaced people in Syria and other conflict-affected countries live precariously in challenging conditions. These conditions will be greatly worsened during localised COVID-19 outbreaks. Further measures are needed to support camp residents to reduce potential COVID-19 transmission, particularly after the first positive COVID-19 cases were reported in OCA on 9 July 2020.

## Declaration of Competing Interest

None declared
